# Features of the monocyte inflammatory response in patients with premature coronary artery disease

**DOI:** 10.52601/bpr.2024.240030

**Published:** 2025-02-28

**Authors:** Tatiana Blokhina, Tatiana Kirichenko, Yuliya Markina, Ulyana Khovantseva, Ivan Melnikov, Olga Guseva, Sergey Bazanovich, Sergey Kozlov, Alexander Orekhov

**Affiliations:** 1 Department of problems of atherosclerosis, Chazov National Medical Research Center of Cardiology of the Ministry of Health of the Russian Federation, 121552 Moscow, Russia; 2 Laboratory of medical genetics, Chazov National Medical Research Center of Cardiology of the Ministry of Health of the Russian Federation, 121552 Moscow, Russia; 3 Laboratory of cellular and molecular pathology of cardiovascular system, State Scientific Center of the Russian Federation Petrovsky National Research Center of Surgery, Moscow 119991, Russia; 4 Laboratory of cell hemostasis, Chazov National Medical Research Center of Cardiology of the Ministry of Health of the Russian Federation, 121552 Moscow, Russia; 5 Laboratory of Gas Exchange, Biomechanics and Barophysiology, State Scientific Center of the Russian Federation, The Institute of Biomedical Problems of the Russian Academy of Sciences, Moscow 123007, Russia; 6 Laboratory of stem cells, Chazov National Medical Research Center of Cardiology of the Ministry of Health of the Russian Federation, 121552 Moscow, Russia

**Keywords:** Coronary artery disease, Monocytes, Inflammatory cytokines, Immune tolerance

## Abstract

The purpose of this study was to examine the secretion of inflammatory cytokines by cultured monocytes/macrophages in patients with premature coronary artery disease (CAD). The study included 38 patients with premature CAD and 35 patients without CAD. A primary culture of CD14+ monocytes was obtained by immunomagnetic separation. The inflammatory response was induced by incubation of a cell culture with lipopolysaccharide (LPS) for 24 hours on Days 1 and 6. Basal and LPS-stimulated secretion of the cytokines, tumor necrosis factor-α (TNF-α), interleukin-1β (IL-1β), interleukin-6 (IL-6), interleukin-8 (IL-8) and monocyte chemotactic protein-1 (MCP-1) was assessed by enzyme immunoassay on Days 2 and 7 of cultivation. The level of basal secretion of TNF-α, IL-1β, IL-6, MCP-1 was higher in patients with CAD compared to patients in the control group. The levels of re-stimulated TNF-α secretion and the levels of LPS-stimulated and re-stimulated IL-1β secretion on the second and sixth days were also higher in patients with CAD. LPS-stimulated MCP-1 secretion on the second day did not differ in patients of both groups, but re-stimulated MCP-1 secretion was higher in patients with CAD. The results of logistic regression analysis showed that the basal secretion levels of IL-1β and IL-6 were independently associated with premature CAD, along with smoking, body mass index and serum HDL-cholesterol levels.

## INTRODUCTION

Conventional cardiovascular risk factors do not always allow timely prediction of the development and course of coronary artery disease (CAD), especially in young patients, when CAD often manifests as myocardial infarction (MI) or sudden cardiac death. In this regard, the identification of new risk factors for premature CAD seems extremely relevant (Le *et al*. 2024)*.* Inflammation is one of the most important mechanisms in the development of atherosclerosis, which underlies CAD. Monocytes are key inflammatory cells that take part in all stages of the development of this process (Dash *et al*. 2024). Pro-inflammatory activation of monocytes in atherosclerosis was previously demonstrated (Nikiforov *et al*. 2019). However, the inflammatory status of monocytes in patients with premature CAD has not been previously studied, which determines the relevance of this study, since the violation of the inflammatory response of monocytes/macrophages contributes to the development of pathologies associated with chronic inflammation, including cardiovascular diseases: CAD, acute MI, atherosclerosis of the carotid arteries (Libby *et al*. 2021). Currently, the identification of the mechanisms of vascular inflammation is of great interest for the development of pathogenetic approaches to the diagnosis and prevention of CAD. The aim of this study was to evaluate the secretion of inflammatory cytokines cultured by monocytes/macrophages in patients with premature CAD.

## RESULTS AND DISCUSSION

The clinical characteristics of study participants are presented in Table 1. Patients with early development of CAD more often suffered from arterial hypertension, and diabetes mellitus, smoked more often, more often had elevated LDL-C levels, and had a higher body mass index (BMI).

**Table 1:** Characteristics of patients.

Parameters	With premature CAD (*n* = 38)	Without CAD (*n* = 35)	*р*
Age, years	54 [50; 55]	52 [46; 59]	0.588
Men/women	30 (79%)/8 (21%)	19 (54%)/16 (46%)	0.05
Arterial hypertension	34 (94%)	19 (54%)	<0.001
Diabetes	15 (42%)	0	<0.001
Smoking	24 (67%)	8 (23%)	<0.001
Family history of CAD	11 (31%)	8 (23%)	0.427
LDL-C >3 mmol/L	35 (92%)	16 (46%)	<0.001
HDL-C <1 mmol/L in men and <1.2 mmol/L in women	21 (55%)	24 (69%)	0.168
Obesity BMI, kg/m^2^	8 (21%) 28 [27; 29]	5 (14%) 26 [24; 28]	0.66 0.005
CAD: oronary artery disease; C: cholesterol, LDL-C: low-density lipoprotein cholesterol; HDL-C: high-density lipoprotein cholesterol; BMI: body mass index. Data are presented as median and first and third quartiles (Me [Q1; Q3]).			

Table 2 presents the results of measuring the concentration of cytokines in the primary culture of monocytes included in the study of patients. Patients with premature CAD compared with patients without CAD had higher basal secretion of tumor necrosis factor-α (TNF-α), interleukin-1β (IL-1β), interleukin-6 (IL-6) and monocyte chemotactic protein-1 (MCP-1). Patients with premature CAD also had higher LPS-stimulated secretion of interleukin-8 (IL-1β) on the second day and TNF-α, IL-1β, MCP-1 on the sixth day. Neither basal nor stimulated IL-8 secretion differed between patients with CAD or without CAD.

**Table 2:** Secretion of inflammatory cytokines by cultured monocytes

Cytokine secretion (pg/mL)	With premature CAD (*n* = 38)	Without CAD (*n* = 35)	*р*
TNF-α			
Basal secretion	228 [216; 239]	192 [138; 213]	<0.001
1 LPS stimulation (2nd day)	3780 [2801; 5882]	3578 [2376; 4694]	0.25
2 LPS stimulation (6th day)	239 [215; 289]	186 [151; 206]	<0.001
IL-1β			
Basal secretion	181 [134; 194]	110 [104; 154]	<0.001
1 LPS stimulation (2nd day)	1536 [968; 1890]	858 [724; 1070]	<0.001
2 LPS stimulation (6th day)	108 [93; 118]	87 [71; 101]	0.001
IL-6			
Basal secretion	327 [303; 451]	301 [271; 370]	0.006
1 LPS stimulation (2nd day)	27879 [8674; 40646]	33397 [26839; 37803]	0.136
2 LPS stimulation (6th day)	1213 [1101; 2114]	1542 [1070; 2077]	0.833
IL-8			
Basal secretion	7057 [4840; 9102]	5910 [4555; 7805]	0.200
1 LPS stimulation (2nd day)	202035 [182065; 212726]	162656 [147135; 212360]	0.09
2 LPS stimulation (6th day)	20111 [12130; 23951]	20217 [12769; 28073]	0.649
МСР-1			
Basal secretion	3092 [2103; 4771]	1923 [1486; 2539]	<0.001
1 LPS stimulation (2nd day)	39240 [16253; 70233]	31526 [20192; 55952]	0.624
2 LPS stimulation (6th day)	10173 [2813; 23221]	2910 [1764; 4097]	0.003
CAD: coronary artery disease; TNF-α: tumor necrosis factor alpha; IL-1β: interleukin-1β; IL-6: interleukin-6; IL-8: interleukin-8; MCP-1: monocyte chemotactic protein-1. Data are presented as median and first and third quartiles (Me [Q1; Q3]).			

Logistic regression analysis was performed to determine indicators independently associated with the presence of premature CAD and assess the severity of the association. Initially, a correlation matrix (not shown) was built to identify indicators associated with the presence of premature CAD. Indicators that demonstrated association with premature CAD were included in univariate logistic regression analysis. Indicators that did not influence the outcome (assignment to the group of patients with CAD) or influenced the outcome indirectly through other factors were excluded from the analysis. Univariate logistic regression analysis included sex, smoking, BMI, hypertension, diabetes mellitus, levels of monocytes, neutrophils, TNF-α, IL-1β, IL-6, MCP-1, and HDL-C as probable predictors of premature CAD. According to the Wald criterion (*p < 0.05*), smoking, BMI, HDL-C, IL-1β and IL-6 levels were included in the multivariate regression model (Table 3). The Hosmer-Lemeshow test for the resulting model was 0.129, Nagelkerke *R*^2^ = 0.557. Thus, the Hosmer-Lemeshow test shows that the observed event rate matches the expected event rate in subgroups of the model population, and the Nagelkerke *R*^2^ shows that the model is acceptable for use.

**Table 3:** Results of logistic regression analysis of independent predictors of the premature CAD

Parameter	β	MSE	Wald criterion	OR (95% CI)	*p*
Smoking	1.427	0.763	3.495	4.166 (0.933−18.594)	0.062
BMI	0.225	0.099	5.15	1.252 (1.031−1.52)	0.023
HDL-C	−1.233	0.589	4.383	0.291 (0.092−0.924)	0.036
IL-1β	0.020	0.009	5.471	1.021 (1.003−1.038)	0.019
IL-6	0.006	0.003	3.698	1.006 (1.001−1.011)	0.054
Constant	−9.835	3.575	7.569	0.000	0.006
BMI: body mass index; HDL-C: high-density lipoprotein cholesterol; IL: interleukin; β: coefficient; MSE: mean square error; OR: odds ratio; CI: confidence interval.					

**Figure 1:**
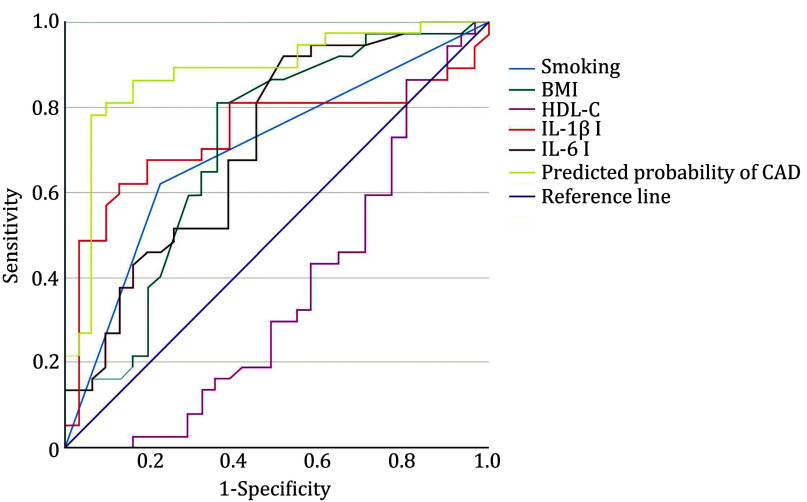
ROC analysis of multivariate logistic regression model. Curves are presented for each of the indicators included in the model separately and for the entire model (predicted probability of having premature CAD)

ROC analysis of the resulting model demonstrated an area under the curve (AUC) of 0.88 ± 0.045 (95% CI 0.79−0.97), *p* < 0.001. The model correctly classified 85.3% of patients with a sensitivity of 90.3% and specificity of 81.1% at a cutoff of 0.61 (Fig. 1).

The causes of early onset of CAD remain unclear and are not determined solely by traditional risk factors. Inflammation plays an important role at all stages of atherosclerosis development. In recent years, the inflammatory response has been intensively studied in regard to the prediction of adverse cardiovascular events. The present study revealed pro-inflammatory activation of monocytes/macrophages in patients with premature CAD, which consists of an increase in basal and induced secretion of inflammatory cytokines TNF-α, IL-1β, MCP-1 and, to a lesser extent, IL-6. The results of logistic regression analysis showed that the levels of IL-1β and IL-6 secretion were independent predictors of premature CAD, along with smoking, body mass index and HDL-C levels.

Studies of associations between interleukin levels and premature CAD are scarce. Experiments in cell culture demonstrated that LPS stimulation increased the secretion of proinflammatory cytokines by cultured monocytes/macrophages and promoted the formation of foam cells (Bekkering *et al*. 2014). IL-1β plays an important role in the pathogenesis of CAD. Production of this cytokine is increased in atherosclerotic plaques, and patients with CAD demonstrate higher concentrations of IL-1β (Kim *et al*. 2020). In addition, IL-1β is involved in atherogenesis by influencing lipid metabolism, activating smooth muscle cell proliferation, and enhancing the procoagulant activity of endothelial cells (Kim *et al*. 2020; Libby *et al*. 1986). The relationship between IL-1β, MI (Iacoviello *et al*. 2005; de Gaetano *et al*. 2011) and stable CAD (Tsimikas *et al*. 2014; Waehre *et al*. 2004) was demonstrated. The CANTOS study demonstrated that an anti-IL-1β monoclonal antibody reduced the risk of adverse cardiovascular events in post-MI patients (Crossman *et al*. 2017). IL-6 was an independent risk factor of acute MI (Ridker *et al*. 2000). High IL-6 levelsare a marker of increased mortality in individuals with unstable CAD, independent of other factors such as troponin T and C-reactive protein (CRP) (Pai *et al*. 2004). TNF-α is involved in atherogenesis through the synthesis of acute phase proteins such as CRP and inflammatory cytokines such as IL-1β and IL-6, and also promotes the recruitment and infiltration of macrophages/monocytes into the arterial subendothelium and the reduction of lipoprotein lipase activity (Henein *et al*. 2022).

During the initiation and progression of atherosclerosis, MCP-1 stimulates monocyte transportation through the endothelium (Deshmane *et al*. 2009). After binding to its monocyte receptor, MCP-1 stimulates monocyte migration to the subendothelial space, which is considered one of the earliest stages of atherogenesis (Amasyali *et al*. 2009). MCP-1 deficiency leads to a sharp decrease in plaque growth. On the contrary, overexpression of MCP-1 by the myeloid compartment is sufficient to aggravate the progression of atherosclerosis (Kim *et al*. 2020). In an analysis of atherosclerotic plaques from 1199 patients undergoing carotid endarterectomy, an association was found between plaque MCP-1 levels and plaque instability. High levels of MCP-1 were associated with higher macrophage content, larger lipid cores, intraplaque hemorrhage, and lower smooth muscle cell and collagen content, *i*.*e*., characteristics of the vulnerable plaque (Georgakis *et al*. 2021b). In a recent study of 8293 individuals, among all 41 cytokines tested, genetically determined levels of MCP-1 showed the strongest association with ischemic stroke, CAD, and MI (Georgakis *et al*. 2019a). In a series of meta-analyses of seven population-based cohorts including 21,401 middle-aged individuals without CAD, higher levels of MCP-1 were associated with ischemic stroke and CAD, as well as cardiovascular mortality. This association was present after adjustment for traditional risk factors and levels of IL-6 and CRP (Georgakis *et al*. 2019b, 2021a).

The results of this study indicate an association of baseline and activated proinflammatory activity of monocytes with CAD.

## MATERIALS AND METHODS

The study included 73 patients: 38 patients with premature stable CAD, including 30 men under the age of 55 years, with manifestation of CAD before 50 years of age, as well as eight women under the age of 65 years, with manifestation of CAD before 60 years of age, in whom coronary angiography (CAG) or computed tomographic angiography (CTA) of the coronary arteries revealed stenotic lesions; 35 patients in the control group, including 19 men under the age of 55 years and 16 women under the age of 65 years, who had no clinical manifestations of CAD and no stenosing coronary atherosclerotic lesions. Indications for performing CAG or CTA of the coronary arteries in patients in the control group were determined by their attending physicians. Coronary atherosclerotic lesions were considered stenotic if they decreased the arterial lumen diameter by 50% or more. Lesions were assessed in the left main, anterior descending, circumflex, right coronary arteries, and second-order branches with a diameter of >2 mm (Neeland *et al*. 2012).

The study did not include men over 55 years of age or women over 65 years of age; patients with familial hypercholesterolemia, low-density lipoprotein cholesterol (LDL-C) level >4.9 mmol/L, unstable angina, in the first two months after myocardial infarction (MI), coronary artery bypass grafting or percutaneous coronary intervention; with the presence of human immunodeficiency virus, hepatitis, syphilis; patients with malignant neoplasms; with clinical and laboratory signs of an acute infectious disease within the previous two months.

All study participants underwent an examination assessing traditional risk factors for CAD, coronary artery CTA, or coronary angiography. The study protocol was approved by the Local Ethics Committee of Chazov National Medical Research Center, Moscow, Russia. The study was performed in accordance with the Declaration of Helsinki of 1964. All participants signed written informed consent to participate in the study.

### Study of the secretion of pro-inflammatory cytokines in primary monocyte culture

Blood was taken from the cubital vein into BD Vacutainer vacuum tubes (Becton Dickinson, USA) containing EDTA (1.6 mg per 1 mL of blood) and the protease inhibitor aprotinin (50 KIU per 1 mL of blood). All experiments were performed within two hours after blood collection.

The level of basal and stimulated secretion of inflammatory cytokines, TNF-α, IL-1β, IL-6, IL-8 and MCP-1 was assessed in primary culture of monocytes taken from patients with CAD and without CAD. The leukocyte fraction of blood cells was obtained from the whole blood of all study participants by centrifugation in a Ficoll gradient, followed by isolation of CD14+ cells using columns for immunomagnetic separation and paramagnetic nanoparticles (Miltenyi Biotec, USA). The isolated cells were cultured in two wells of a culture plate at a rate of 500,000 cells per well in 0.5 mL of X-VIVO culture medium (Lonza, Germany) in a CO_2_ incubator at 37 °C for 24 hours. mCSF was added into the culture medium at a concentration of 50 ng/mL. Monocytes differentiated into macrophages on Day 7, the content of CD68+ cells after the differentiation period was 90%–95%. In the first well, basal secretion of inflammatory cytokines by cultured monocytes was assessed. Bacterial lipopolysaccharide (LPS) was added to the second well at a concentration of 1 μg/mL to stimulate the inflammatory response. Culture fluid samples were obtained after 24 hours to determine spontaneous and LPS-stimulated secretion of inflammatory cytokines. After five days of rest, repeated LPS stimulation of the cells of the second well was performed for 24 hours to assess the tolerance of the immune response. The concentration of inflammatory cytokines in culture fluid samples was determined by enzyme immunoassay using commercial DuoSet ELISA kits (R&D Systems, USA). The level of inflammatory cytokines secretion by monocytes from CAD patients is assessed in comparison to the monocytes of control patients without CAD that allows not to consider inflammatory pre-activation of monocytes during isolation as a limitation of the study.

### Statistical analysis

Statistical data analysis was performed using SPSS Statistics v. 27.0 (SPSS Inc., USA) and Statistica v. 6.0 (StatSoft Inc., USA). The collected quantitative data are presented as mean and standard deviation, as well as median and quartiles (25th and 75th percentiles). To test statistical hypotheses about the type of distribution, the Shapiro-Wilk’s W test was used. For a comparative analysis of data from patients in both groups, nonparametric statistical methods were used: Fisher’s exact test and the *χ*² test with Yates’ correction when comparing qualitative characteristics, and the Mann-Whitney U test when comparing quantitative characteristics in two independent groups. Logistic regression analysis was used to detect independent risk factors associated with an increased likelihood of CAD in men under 55 years of age and in women under 65 years of age. Indicators included in the regression model were determined using the Wald test. To assess the quality of the model, the Hosmer-Lemeshow goodness-of-fit test, Nagelkerk R2 and ROC analysis were used. The results of the ROC analysis, as well as the Youden test, were used to calculate the optimal cut-off point.

## Conflict of interest

Tatiana Blokhina, Tatiana Kirichenko, Yuliya Markina, Ulyana Khovantseva, Ivan Melnikov, Olga Guseva, Sergey Bazanovich, Sergey Kozlov and Alexander Orekhov declare that they have no conflict of interest.
